# Developmental Plasticity of the Amphibious Liverwort *Riccia fluitans*

**DOI:** 10.3389/fpls.2022.909327

**Published:** 2022-05-23

**Authors:** Felix Althoff, Linus Wegner, Katrin Ehlers, Henrik Buschmann, Sabine Zachgo

**Affiliations:** ^1^Department of Botany, Osnabrück University, Osnabrück, Germany; ^2^Department of Botany, Justus-Liebig University, Gießen, Germany; ^3^Department of Molecular Biotechnology, University of Applied Sciences Mittweida, Mittweida, Germany

**Keywords:** *Riccia fluitans*, adaptation, terrestrialization, rhizoids, air pores, cuticle

## Abstract

The colonization of land by ancestors of embryophyte plants was one of the most significant evolutionary events in the history of life on earth. The lack of a buffering aquatic environment necessitated adaptations for coping with novel abiotic challenges, particularly high light intensities and desiccation as well as the formation of novel anchoring structures. Bryophytes mark the transition from freshwater to terrestrial habitats and form adaptive features such as rhizoids for soil contact and water uptake, devices for gas exchange along with protective and repellent surface layers. The amphibious liverwort *Riccia fluitans* can grow as a land form (LF) or water form (WF) and was employed to analyze these critical traits in two different habitats. A combination of light microscopy, scanning electron microscopy (SEM) and transmission electron microscopy (TEM) studies was conducted to characterize and compare WF and LF morphologies. A complete phenotypic adaptation of a WF plant to a terrestrial habitat is accomplished within 15 days after the transition. Stable transgenic *R. fluitans* lines expressing GFP-TUBULIN and mCherry proteins were generated to study cell division and differentiation processes and revealed a higher cell division activity in enlarged meristematic regions at LF apical notches. Morphological studies demonstrated that the *R. fluitans* WF initiates air pore formation. However, these pores are arrested at an early four cell stage and do not develop further into open pores that could mediate gas exchange. Similarly, also arrested rhizoid initial cells are formed in the WF, which exhibit a distinctive morphology compared to other ventral epidermal cells. Furthermore, we detected that the LF thallus has a reduced surface permeability compared to the WF, likely mediated by formation of thicker LF cell walls and a distinct cuticle compared to the WF. Our *R. fluitans* developmental plasticity studies can serve as a basis to further investigate in a single genotype the molecular mechanisms of adaptations essential for plants during the conquest of land.

## Introduction

Terrestrial colonization is one of the most significant evolutionary episodes in Earth history. Land plants evolved from an ancestral freshwater streptophycean alga and the origin of extant land plant lineages dates back ca 470 MYA (de Vries and Archibald, 2018; Morris et al., 2018). Colonization of land by plants transformed our planet Earth and also changed the evolutionary trajectories of other lineages of life. Novel ecological niches were formed, necessitating morphological and metabolic plant adaptations for a life, lacking buffering aquatic capacities (Delwiche and Cooper, 2015; Rensing, 2020). By the early Silurian, land plants had evolved a series of crucial innovations such as adaptative devices for gas exchange, water and nutrient uptake as well as protective cell surface layers (Morris et al., 2018). Advances in *Marchantia polymorpha* research, driven by development of a comprehensive molecular tool box, enabled characterizing key regulators and molecular processes controlling these adaptive features (reviewed by Ishizaki et al., 2016; Kohchi et al., 2021; Romani and Moreno, 2021). Recently, an *Agrobacterium*-based transformation method has been established for the amphibious liverwort *R. fluitans*, whereby stable transgenic lines can be generated in about 10 weeks (Althoff and Zachgo, 2020). Amphibious plants can grow either being submerged under water or exposed to air and thus offer to study environmental adaptations regulated by a single genotype. *Riccia* is the most species-rich genus in complex thalloid liverworts (Söderström et al., 2016; Villarreal et al., 2016). The evolution of phenotypic plasticity in *R. fluitans*, where the amphibious lifestyle is likely a secondary adaptation, might have been supported by a faster evolution rate in this genus (Villarreal et al., 2016).

Two axenic *R. fluitans* cultures, the ecotypes 001TC and BoGa, are available. *R. fluitans* 001TC is a commercial line. The water form (WF) produces only small intercellular spaces and plants thus thrive fully submerged close to the ground (Andersen and Pedersen, 2002; Althoff and Zachgo, 2020). The BoGa ecotype was recently established (Althoff and Zachgo, 2020) and here, the WF develops larger intercellular spaces mediating floating of thalli below the water surface. A crucial role for the hormone abscisic acid (ABA), stimulating aquatic leaf formation in amphibious angiosperms as well as water-to-land thallus transitions of *R. fluitans*, has been demonstrated (Hellwege et al., 1992; Koga et al., 2021). Whole genome duplications correlate with environmental changes and stress, which are considered as major drivers of advancing plant complexity (Van de Peer et al., 2017). The amphibious *Riccia rhenana* liverwort, morphologically resembling *R. fluitans*, comprises 16 chromosomes. *R. fluitans* only possesses eight chromosomes and the absence of a whole genome duplication makes it eligible for further molecular studies (Wyatt and Davison, 2013; Althoff and Zachgo, 2020).

The amphibious life style of *R. fluitans* enables investigations of distinctive morphological features between the WF and land form (LF), particularly of rhizoid and air pore formation, crucial for the evolution of land plants. Whereas smooth rhizoids are also formed by simple thalloids, only complex thalloid liverworts additionally develop pegged rhizoids (Duckett et al., 2014). This striking feature raised the attention of botanists already in the 19th century (Leitgeb, 1881; Kny, 1890; Kamerling, 1897). Later, it was shown that mature smooth rhizoids are alive and mediate nutrient uptake and endophytic entry as well as anchorage to the soil. Differently, pegged rhizoids are dead at maturation and capillary strands oriented parallel to the thallus serve as an external water-conducting system (Goebel, 1930; McConaha, 1941; Pocock and Duckett, 1985; Duckett et al., 2014). The bHLH transcription factor Mp*RSL1* is a key regulator of rhizoid formation in *M. polymorpha* and its activity is directly repressed by the microRNA Mp*FRH1* (Proust et al., 2016; Thamm et al., 2020).

Complex *M. polymorpha* air pores are composed of a multilayered, 16-cell barrel-shaped structure forming an opening in the dorsal epidermal cell layer to facilitate gas exchange between the underlaying air chamber with photosynthetic filaments and the atmosphere (Barnes and Land, 1907; Apostolakos et al., 1982). Intercellular spaces are generated during air chamber growth by schizogenic cell separation (Ishizaki, 2015). In *M. polymorpha*, air pore and air chamber formation mediating gas exchange is controlled by the Plant U-box (PUB) gene Mp*NOP1* and the WIP zinc-finger transcription factor Mp*WIP* (Ishizaki et al., 2013; Jones and Dolan, 2017). *R. fluitans* LF plants form open, simple air pores (Villarreal et al., 2016). Air pore development in *R. fluitans* WF plants has been less well investigated and did not reveal formation of fully developed open air pores (Klingmüller and Denffer, 1959).

Another evolutionary key adaptation of embryophytes to a terrestrial life was the formation of a cell wall with a hydrophobic cuticle on arial plant surfaces limiting transpiration as well as uncontrolled water absorption (Raven, 2002; Philippe et al., 2020). Recent investigations started to unravel the underlying genetic pathways in liverworts. Mp*SBG9*, a MYB transcription factor, is a key regulator of cutin formation in *M. polymorpha*, implying conserved regulatory MYB MIXTA-like transcription factor functions in cuticle regulation during land plant evolution (Xu et al., 2021). A large diversity of cell wall polymers and cuticle compounds contributed to cope with variable, often limited water availability (Sørensen et al., 2010; Philippe et al., 2020). The production of cuticular waxes in *M. polymorpha* is affected by growth conditions, which together with cell wall polymer diversity likely contributed to the adaptation to terrestrial habitats (Kolkas et al., 2021; Xu et al., 2021).

The aim of the present study was to take advantage of the amphibious *R. fluitans* liverwort to characterize crucial adaptive features in WF and LF plants by different microscopy techniques. Transgenic GFP-TUBULIN and mCherry expressing lines were generated to investigate cell division and differentiation processes in the aquatic and terrestrial growth forms. Recent advances in *M. polymorpha* research uncovered key regulators controlling processes like air pore and rhizoid formation and the here described developmental plasticity of *R. fluitans* can be exploited to further unravel adaptive molecular mechanisms during terrestrialization.

## Materials and Methods

### Plants and Growth Conditions

*R. fluitans* ecotype BoGa derived from a pond in the Botanical Garden of the Osnabrück University was used to establish an axenic culture (Althoff and Zachgo, 2020). LF thalli were grown on solid half strength Gamborg’s B5 medium with vitamins (1/2 GB5; Duchefa, Haarlem, The Netherlands) supplemented with 14 g/L agar-agar Kobe I (Roth, Karlsruhe, Germany) in 10 × 10 cm petri dishes (Sarstedt, Nümbrecht, Germany). WF thalli were cultivated in eco2boxes (Duchefa, Haarlem, The Netherlands) with 4 ml of autoclaved scaper’s soil (Dennerle GmbH, Münchweiler an der Rodalb, Germany) and 200 ml of liquid 1/2 GB5 medium, where pH 6 was adjusted with 5 N KOH. Plants were grown in climate chambers at 22°C under long-day conditions (16 h light; 8 h dark) equipped with cool white (840) fluorescence bulbs emitting 60 μmol m^−2^ s^−1^ photons.

### Generation of Transgenic *Riccia fluitans* BoGa Lines

The *pro*Mp*EF1α*:*GFP*-Mp*TUB1* construct (Buschmann et al., 2016) was used as cell division marker and transformed into *R. fluitans* BoGa based on the recent *R. fluitans* transformation protocol with few modifications. In addition, mCherry reporter lines were generated by transforming *pro*Mp*EF1α*:*mCherry* into *R. fluitans* BoGa (Althoff and Zachgo, 2020). Thallus tissue from *R. fluitans* BoGa was razor blade chopped, distributed on 1/2 GB5 solid medium plates containing 400 μM NAA and cultivated for 4  weeks to generate callus tissue. Calli were collected, razor blade chopped and transferred into liquid cultures for transformation. The pGWB6-GFP-MpTUB1 and pMpGWB-mCherry vectors were transformed into *Agrobacterium tumefaciens* C58C1 (pGV2260) by electroporation and a single colony of each construct was inoculated into fresh LB medium supplemented with 100 μg/ml rifampicin, 100 μg/ml carbenicillin, 100 μg/ml kanamycin, 50 μg/ml hygromycin and used for transformation procedure. Co-culture and selection of transgenic *R. fluitans* BoGa using hygromycin (5 and 10 μg/ml) and cefotaxime (100 μm/ml) was carried out as described by Althoff and Zachgo (2020). Ten transgenic *R. fluitans* BoGa lines expressing GFP-MpTUB1 and several mCherry lines survived a second selection step with 10 μg/ml hygromycin and were analyzed for fluorescence signals. All examined lines expressed GFP or mCherry and two lines for each construct were chosen for further analyses.

### Light and Transmission Electron Microscopy

For light and transmission electron microscopy (LM, TEM), samples were prepared as described by Venneman et al. (2020) with modifications. About 30 branched plant apices of approx. 6–10 mm length were harvested from axenic *R. fluitans* BoGa LF and WF cultures and embedded in between two layers of 2% (w/v) low gelling agarose at 40°C (type VII; Sigma-Aldrich, Steinheim, Germany). Solidified agarose blocks with LF samples were fixed for 2 h in 50 mM phosphate buffer containing 2% (v/v) glutardialdehyde (GA) at pH 7.2 and RT, and another 2 h in fresh fixation solution on ice. For WF samples, the fixation buffer contained only 1.5% GA. Subsequently, samples were washed in 50 mM phosphate buffer (pH 7.2), and post-fixed overnight in 0.9% (w/v) OsO_4_ in 100 mM phosphate buffer at 4°C. After rinsing in water, samples were stained for 2 h with 0.5% (w/v) aqueous uranyl acetate on ice, dehydrated in a graded ethanol series and propylene oxide and embedded in Spurr’s epoxy resin (adapted mixture; Spurr, 1969). Polymerization was performed at 68°C for 24 h.

For LM analyses, semithin serial sections of about 1 μm thickness were cut on a Reichert Om U2 ultramicrotome (Leica Microsystems GmbH, Wetzlar, Germany) with glass knifes, stained with crystal violet and examined with a Leica DM 5500 microscope B, which was equipped with a Leica DFC 450 camera (Wetzlar, Germany) driven by the Leica Application Suite software (Version 4.3.0). For TEM, serial ultrathin sections were cut with a diamond knife, mounted on Formvar-coated single-slot copper grids, and stained with 2% (w/v) uranyl acetate (12 min) and lead citrate (12 min) (Reynolds, 1963). Examination was performed with an EM912AB transmission electron microscope (Zeiss, Oberkochen, Germany) at 120 kV accelerating voltage under zero-loss energy filtering conditions. Images were recorded with a 2 k × 2 k dual-speed slow-scan CCD camera (SharpEye, TRS, Moorenweis, Germany) using the iTEM software package (OSIS).

Transmission electron microscopy micrographs were taken from at least 30 individual upper epidermal cells of each of three independent samples of the LF and the WF, respectively. Micrographs were used for calibrated measurements of the thickness of the outer cell wall.

### Scanning Electron Microscopy

For scanning electron microscopy (SEM), LF and WF thallus apices were transferred to fresh agar plates or liquid medium and cultivated for 2–3  weeks. WF and LF tissues were then fixed with a FAE fixative containing 4% formaldehyde, 5% acetic acid, and 50% ethanol. To examine the transfer form, at least 16 WF thalli for every time point were transferred to solid agar plates and fixed after 3, 6, 9, 12, and 15 days in FAE. Tissues were incubated for 2 days at 4°C in the FAE fixative and afterward washed and dehydrated in an ethanol series. Tissues were critical point dried in a Leica EM CPD300 device (Wetzlar, Germany) applying 36 CO_2_ changes. Dried samples were mounted on a pin stub such that different ventral and dorsal sides could be investigated and sputtered with gold in a Leica EM ACE600 sputter coater (Wetzlar, Germany). Around 12 nm gold coat was applied during two runs in 0° and 15° angles. Tissues were analyzed with a Jeol JSM-IT200 (Freising, Germany) at 10–15 kV.

For epidermal cell length measurements of dorsal thallus sides, four individual thalli were each analyzed for WF and LF. Epidermal cell length of 10 cells in five areas with distances of 0–100, 100–200, 200–300, 300–400, and 400–500 μm from the apical notch tip were measured per thallus.

### Confocal Laser Scanning Microscopy (Airy Scan)

Transgenic lines expressing the microtubule marker GFP-MpTUB1 or mCherry were transferred to fresh solid or liquid medium 3 days before analysis. Plants were imaged with a Zeiss LSM 880 confocal microscope (Oberkochen, Germany) using the 20x objective for overview scans and the 63x objective for high resolution images. GFP was imaged using the argon laser (488 nm; combined with a band pass filter) and mCherry was excited by a diode laser (DPSS at 561 nm; combined with appropriate filters). Chlorophyll autofluorescence was recorded simultaneously with GFP (excited by the argon laser). Images were further processed using the Fiji freeware (version 2.3.0/1.53f; Schindelin et al., 2012).

### Measurements of Thallus Width

To measure thallus width in WF and LF at thallus apices and more posterior positions, thallus apices were transferred to fresh agar plates (LF) or culture boxes with liquid medium (WF) and grown for 14 days. Pictures from individual plants were taken with the Leica M165 FC stereo microscope and a Leica DFC490 camera (Wetzlar, Germany). WF thalli were transferred from the liquid medium to a solid agar plate and contorted thalli were spread and flattened for picture taking. Width of thallus apices of 10 WF and 10 LF plants were measured, resulting in analysis of 123 WF and 109 LF apices. Thallus width at the posterior region was measured on 15 WF and LF plants each, resulting in 60 measured regions per growth form.

### Analyses of Thallus Surface Permeability

WF and LF thalli were harvested from 3–4 weeks old cultures and transferred into a 50 ml falcon tube containing 45 ml 100% ethanol. Samples were incubated on a horizontal shaker at room temperature. Thalli were removed after 5, 10, 20, 30, 60, 90, and 120 min and washed in ddH_2_O to stop the chlorophyll bleaching reaction. Overview pictures were taken with a Canon EOS 750D DSLR camera and detailed pictures of individual thalli with a Leica M165 FC stereo microscope equipped with a Leica DFC490 camera (Wetzlar, Germany).

### Statistical Analysis

Measurements were performed with Fiji (version 2.3.0/1.53f; Schindelin et al., 2012) or ImageJ (version v1.53e; Schneider et al., 2012) image processing program measurement tools. Statistical analysis and diagrams were conducted with GraphPad Prism 9 (version 9.3.1). Sample sizes and biological replicate numbers are specified in each figure legend and values indicated in the text are means with SD. Welch’s *t*-test or one-way ANOVA corrected by Turkey analysis was performed for statistical significance as indicated in the respective figures by ^**^p < 0.01, ^***^p < 0.001, and ^****^p < 0.0001.

## Results

### Morphological Analysis of *Riccia fluitans* WF and LF Development

Recently, we established a transformation strategy for the *R. fluitans* ecotype 001TC, a popular aquarium plant that has been commercially available for a long time (Andersen and Pedersen, 2002; Althoff and Zachgo, 2020). Here, we are using the BoGa ecotype for all analyses, as it shows a more vigorous growth under our culture conditions.

**Figure 1:**
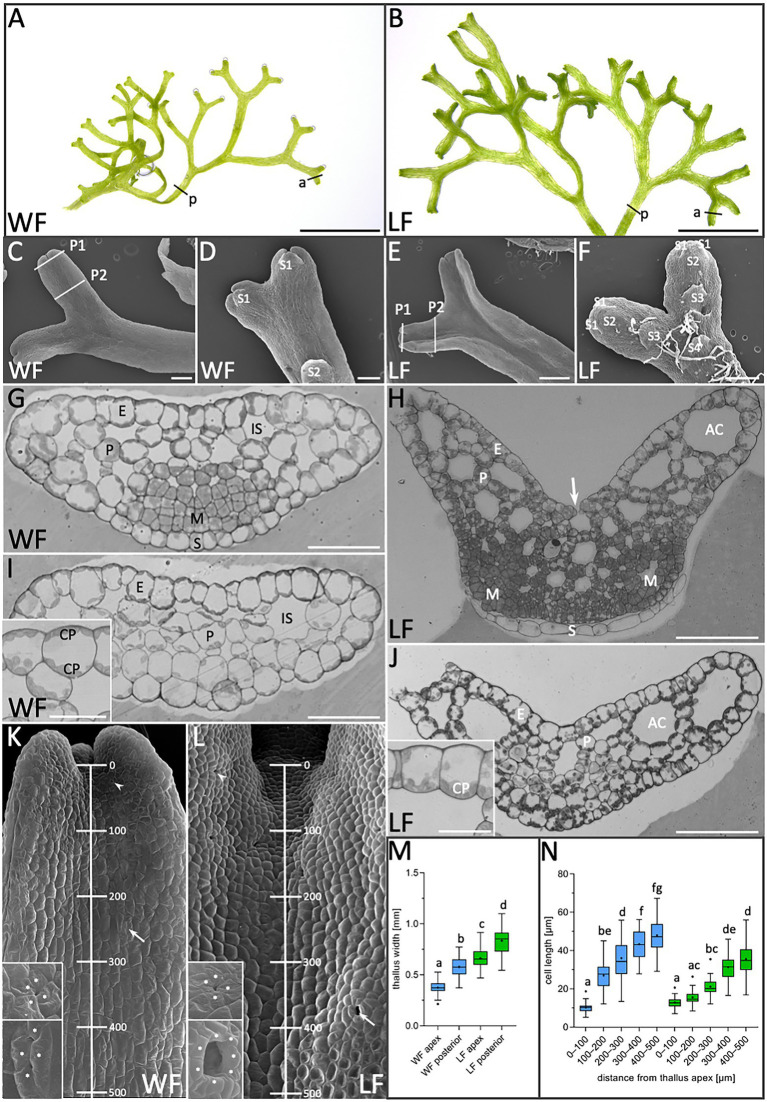
Morphological comparison of *R. fluitans* water form (WF) and land form (LF) thalli. **(A)** WF and **(B)** LF plant habitus. **(C)** WF dorsal, **(D)** ventral thallus side and **(E**,**F)** respective LF thallus sides. P1, P2: Sectioning planes of younger meristematic (P1) and older differentiated thallus tissue (P2). Positions of the youngest (S1) and successively older ventral scales (S2, S3, and S4) are indicated. **(G**,**H)** WF and LF cross sections through P1 and **(I**,**J)** through P2. Insets in **(I**,**J)** show details of epidermal cells. Arrow in **(H)** indicates an air pore opening. **(K**,**L)** Dorsal view on WF and LF thalli indicating the 500 μm range from the apical notch toward the posterior thallus used for cell length quantification. Arrowheads point to young air pore invaginations (upper insets) and arrows to an arrested older WF pore (lower inset) in **(K)** and a mature, open LF pore (lower inset) in **(L)**. Pore ring cells are marked by asterisks according to probable cell borders. **(M)** Quantification of thallus width at WF (blue) apices (a in A; *n* = 123, 10 plants) and posterior regions (p in A; *n* = 60, 15 plants). LF (green) apex (a in B; *n* = 109, 10 plants) and posterior regions (p in B; *n* = 60, 15 plants). Letters above columns indicate *p* < 0.0001. **(N)** Quantification of WF (blue) and LF (green) cell length (*n* = 50 cells, four thalli/form, *p* < 0.0001) in the five regions indicated in **(K**,**L)**. E, epidermis cell; P, parenchyma cell; IS, intercellular space; AC, air chamber; M, meristematic cells; S, ventral scale; and CP, chloroplasts. Scale bars: **(A,B)** 5 mm; **(C,D,F)** 200 μm; **(E)** 500 μm; **(G,I)** 50 μm; **(H,J)** 100 μm; and (**I,J** insets) 25 μm.

*R. fluitans* BoGa WF and LF thalli were compared regarding their overall morphological differences. WF plants grow below the water surface and their free-floating thalli appear thinner than LF thalli (Figures 1A,B). Quantification of thallus width in apical notches, regions with a high cell division activity, and in more posterior regions, comprising differentiated cells (Figures 1A,B,a,p), confirmed these observations. The average width of WF thalli is 375 ± 58 μm in apices and 552 ± 81 μm in posterior regions, whereas corresponding values for LF thalli are 662 ± 94 μm and 829 ± 147 μm, respectively (Figure 1M). Compared to the WF, the LF thus forms thalli with an approximately 1.5-fold increased width. A difference was also observed concerning the formation of rhizoids, single-celled structures mediating nutrient uptake and anchoring plants to the soil. WF thalli show an almost complete absence of rhizoids (Figure 1D). Differently, LF thalli develop numerous rhizoids, which are initiated in central thallus regions (Figure 1F). Ventral scales are regularly formed, with larger distances in WF compared to LF thalli (Figures 1D,F).

Next, cross sections were made through the apex (Figures 1G,H) and posterior thallus regions (Figures 1I,J) as indicated by section plains P1 and P2 in Figures 1C,E, to compare inner thallus morphologies. Thalli of both growth forms are composed of a single-layered epidermis encompassing parenchyma cells. Smaller intercellular spaces are interspersed in WF parenchymatic tissue (Figures 1G,I), whereas larger intercellular spaces are formed in the LF (Figures 1H,J). The latter are indicated as air chambers, which are connected to the environment *via* air pores (Apostolakos et al., 1982; Shimamura, 2016). Notably, WF chloroplasts are quite evenly distributed on both periclinal epidermis cell walls (Figures 1G,I inset). In contrast, LF chloroplasts accumulate on basal epidermis cell walls facing the air chambers (Figures 1H,J inset) and are preferentially localized in parenchyma cells toward air chambers, suggesting that these differences can contribute to physiological adaptation mechanisms. Air pores are initiated on dorsal thallus sides of both *R. fluitans* forms by four pore ring cells (Figures 1K,L upper insets). In WF thalli, initial air pore formation generates a small pore invagination. During further cell growth processes, protrusions from two opposing pore ring cells extend toward the center of the invagination, hindering water entrance (Figure 1K, lower inset). Development of open, mature air pores was only observed in LF thalli (Figure 1L, lower inset).

In both growth forms, small, undifferentiated cells were detected in the ventral region of cross sections through apices, which indicate meristematic zones with high cell division activity (Figures 1G,H). As WF and LF thalli both undergo bifurcation events (Figures 1A,B), cross sections revealed either single meristematic regions (Figure 1G) or two meristematic regions on ventral thallus sides, the latter representing more advanced stages, where the bifurcation process had already started (Figure 1H). Serial sectioning showed that small meristematic cells of both growth forms are shielded by scales that grow parallel to the ventral thallus surface and consist of a single cell layer with a different morphology (Figures 1G,H). Small, meristematic cells are restricted to WF and LF thallus apices and larger, differentiated cells were detected in posterior thallus regions (Figures 1I,J).

Using SEM, we next determined whether WF and LF thalli differ in the distribution of meristematic and differentiating cells. Cell length was measured starting in the dorsal thallus apex region and moving in five consecutive 100 μm steps gradually downward (Figures 1K,L,N). In the region between 0 and 100 μm, WF cell length was 12.9 ± 2.7 μm and for LF 10.0 ± 2.5 μm. Notably, in the region between 100 and 200 μm, WF cells strongly elongated and increased their length to 27.0 ± 7.6 μm, while LF cells revealed a less severe increase up to 15.6 ± 3.4 μm. Thereafter, WF as well as the LF cells expanded more steadily and reached in the 400–500 μm region a length of 47.9 ± 9.2 μm for the WF and of 36.2 ± 9.5 μm for the LF. These measurements reveal an impact of the two different growth conditions on the dynamics of the transition from cell division to cell differentiation processes and the final length of differentiated cells.

### Analysis of *Riccia fluitans* WF Adaptation to a Terrestrial Lifestyle

**Figure 2:**
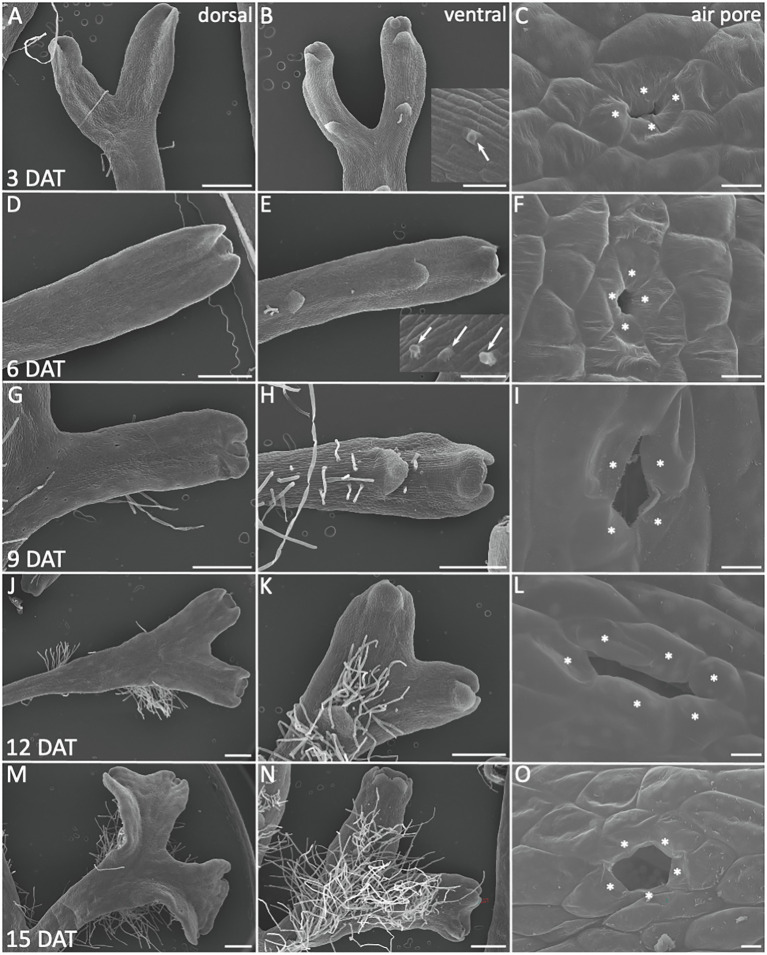
Morphological adaptations during *R. fluitans* WF to LF transition. WF thalli were transferred to solid medium and investigated for morphological changes 3, 6, 9, 12, and 15 days after transfer (DAT). Left column shows dorsal, middle column ventral thallus sides, the latter revealing scale and rhizoid formation. Air pore structures are shown in the right column. **(A**–**C)** 3 DAT. Arrow in inset **(B)** points to outgrowth from a rhizoid initial cell. **(C)** WF air pore with small opening, likely by collapse of formerly invagination covering protrusions. **(D–F)** 6 DAT. Inset in **(E)** shows outgrowing rhizoids (arrows). **(G**–**I)** 9 DAT. **(J–L)** 12 DAT. **(M–O)** 15 DAT, a typical mature, large air pore, formed by more than the initial four pore ring cells **(O)**. Pore ring cells are marked by asterisks according to probable cell borders. Scale bars: 500 μm; except for **(C,F,I,L,O)**: 10 μm.

**Figure 3:**
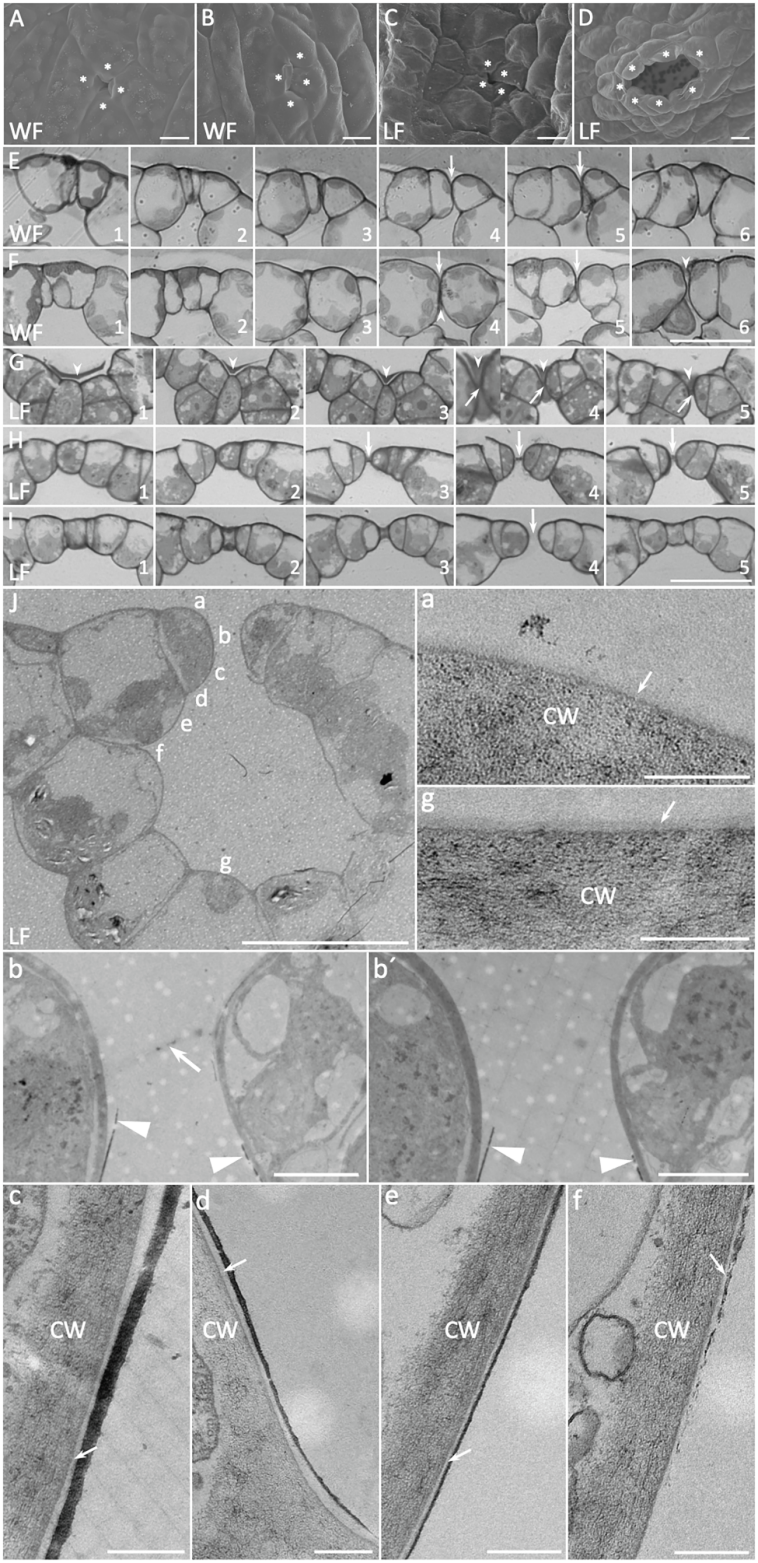
Development of *Riccia fluitans* WF and LF air pores. **(A**,**C)** Young WF and LF air pore invaginations in the apical notch region are formed by four initial air pore ring cells. **(B)** Arrested older WF air pore in a posterior thallus region, covered by protrusions of two opposing pore ring cells, whereas **(D)** shows a mature LF air pore in the respective position. **(E)** Serial cross sections through an arrested WF air pore invagination (arrows), where rounded ring cells adjoin each other. **(F)** Cross section of four different (F1–F2, F3–F4, F5, and F6) arrested WF air pores. Arrowheads indicate extracellular plugs, occluding extremely narrow pore openings underneath pore invaginations (arrows). **(G)** Serial cross sections through a young LF air pore invagination reveal a darker extracellular mass (arrowheads) covering the pore invagination, where opposing pore ring cells are still connected by a thin bar-shaped structure (arrows) traversing a developing central pore. **(H**,**I)** Serial cross sections through an intermediate pore stage **(H)** and an open **(I)** LF air pore. The pore is still sealed by the bar-shaped structure in the median plain in (**H**; arrows) and already open in (**I**; arrow). **(J)** Cross section through a mature LF air pore and air chamber with cell wall and additional layer details, depicted in (a–g). (a,g) Show outer periclinal walls of a pore ring cell and a bottom air chamber cell, respectively, forming an additional layer on the cell wall, likely representing the cuticle (arrows). Arrow in (b) points to remnants of a bar-shaped structure still spanning the pore, similar as observed in H4, which is absent in another serial section of the same pore cut in a median plane (b′). A massive dark layer, likely representing epicuticular waxes is deposited on the lower part of the anticlinal pore ring cell walls, facing the pore (b,b′ arrowheads) and sometimes detaches at the tip. (c–f) Show the progression of the layer along the pore ring cells toward the inner lumen of the air chamber. Arrows point to the cuticle between cell walls and wax layer. Pore ring cells are marked by asterisks according to probable cell borders. Scale bars: **(A–C)** 10 μm; **(D)** 20 μm; **(E–I)** 25 μm; **(J)** 20 μm; **(J)** a,c–g 200 nm; and **(J)** b,b′ 2 μm.

Next, we aimed to determine the time period required for a full morphological adaption from an aquatic to terrestrial lifestyle and investigated the morphology of transition stages (Figure 2). *R. fluitans* BoGa WF thalli were transferred to solid medium and processed for SEM analyses of dorsal and ventral thallus surfaces every 3 days till 15 days after transfer (DAT). A narrow air pore opening between the four pore ring cells was observed three DAT (Figure 2C). We assume that such pores were initiated and arrested in their development before the transfer occurred. Thereafter, a collapse of ring cell protrusions covering the pore invagination likely formed a small opening. Six DAT, we observed pores with enlarged pore openings (Figure 2F) that further increased in size throughout nine and 12 DAT (Figures 2I,L). These pores likely still represent modifications of WF pores as their pore sizes still remained smaller than pores formed in LF thalli (Figure 1L). About 15 DAT, pores then resembled LF air pores (Figure 2O). Formation of mature, fully opened LF pores thus likely requires novel initiation of air pores from meristems that have already been transferred to land. We noted a variable increase in the number of pore ring cells, suggesting a participation of cell division activities in pore opening (Figure 1L lower inset, Figures 2L,O). First rhizoid initiation steps in the ventral apical region were visible three DAT by the formation of cellular protrusions from elongated ventral epidermal cells (Figure 2B, inset). About 6 DAT, more rhizoids were initiated and further outgrowth was observed (Figure 2E, inset). The number and length of rhizoids steadily increased during the following days and they were initiated closer to the apices, resulting 15 DAT in the formation of a dense rhizoid network (Figures 2H,K,N). Compared to 3 DAT (Figures 2A,B) and 6 DAT (Figures 2D,E) an increase in thallus width in the apical region started to be visible 9 DAT (Figures 2G,H) and became more pronounced 12 DAT (Figures 3J,K). Finally, 15 DAT the typical LF thallus width was accomplished (Figures 1M, 2M,N). The developmental plasticity of *R. fluitans* thus enables this amphibious liverwort to adapt within 15 days after a water-to-land transition to a novel terrestrial environment.

### Air Pore Development in *Riccia fluitans* WF and LF

LF thalli form simple air pores for gas exchange and SEM analyses showed that they are initiated in thallus apices by an invagination between the four pore ring cells (Figures 1L, 3C). These early invaginations between four pore ring cells were also detected in the apex region of WF thallus (Figures 1K, 3A). WF and LF air pore formation was compared by serial sections through different air pore developmental stages (Figures 3A–D). In differentiating LF apical regions, open air pores are already present (Figure 3I4). Comparable WF thallus positions show that rounding of air pore ring cells contributes to form an invagination (Figures 3E,F arrows) and sometimes produces an extremely narrow gap sealed by an extracellular mass (Figure 3F4, 6 arrowheads).

Analyses of LF pores at young, intermediate and older stages (Figures 3G–I) showed that air pores were formed by small pore ring cells, which are overlayed by an extracellular mass (Figure 3G arrowheads). The small gap between the young pore ring cells that can be seen in median sections was initially plugged by a central bar-shaped structure (Figure 3G4, 5 arrows) and covered by an extracellular mass (Figure 3G4, 5 arrowheads). During further LF pore development, pore ring cell divisions, cell wall rounding and enlargement of the underlying air chamber likely mediate separation of pore ring cells. A remaining central bar-shaped structure, still present in median planes of a medium staged air pore (Figure 3H3–5 arrows), seems to be stretched out until it finally disappears, generating an opened pore (Figure 3I arrow). LF air pores were also examined by TEM of ultra-thin cross sections (Figure 3J), with a focus on cell surfaces bordering the pore opening and their neighboring cells. Images with different upper as well as lower air pore cell wall details are shown in Figures 3Ja–g. Closely before final pore opening, remnants of the central bar-shaped structure, spanning the pore invagination in peripheral (Figure 3Jb arrow) but not median section planes (Figure 3Jb′) were still occasionally detectable. These findings further support observations from Figures 3G–I that the bar-shaped structure is pulled apart during pore maturation and thereby generates an open pore.

Furthermore, TEM analyses revealed the presence of a more translucent and thus likely cuticular layer, covering the cell walls of LF pore ring cells (Figure 3Ja) and the adjacent air chamber cells (Figure 3Jg). Interestingly, we detected an additional, massive darker layer, which likely represents epicuticular waxes. This layer is formed at the narrowest point of the pore ring cells just below the bar-shaped structure (Figures 3Jb,b,′ arrowheads), from where it extends and tapers further downward (Figures 3Jc–e) before it fully disappears at the adjoining cell below the pore ring cell (Figure 3Jf). The spatially highly restricted deposition of presumably hydrophobic waxes suggests a contribution to reduce water infiltration *via* open LF *R. fluitans* pores.

### Characterization of *Riccia fluitans* Rhizoid Formation

Transgenic *R. fluitans* BoGa lines expressing GFP fusions with TUBULIN1 from *M. polymorpha* (GFP-MpTUB1) and the reporter mCherry were investigated by confocal laser microscopy. Both proteins were expressed under control of the *_pro_*Mp*EF1α* promoter from *M. polymorpha* (Althoff et al., 2014), which was shown to drive strong mCherry expression in the cytoplasm and nuclei of *R. fluitans* 001TC thalli (Althoff and Zachgo, 2020). GFP-labeled MpTUB1 is a marker for microtubules and has been applied for cell division analyses in *M. polymorpha* (Buschmann et al., 2016). Several transgenic *R. fluitans* BoGa plants, all strongly expressing GFP-MpTUB1 or mCherry, were generated and further studies were conducted with two GFP-MpTUB1 and two mCherry lines.

**Figure 4:**
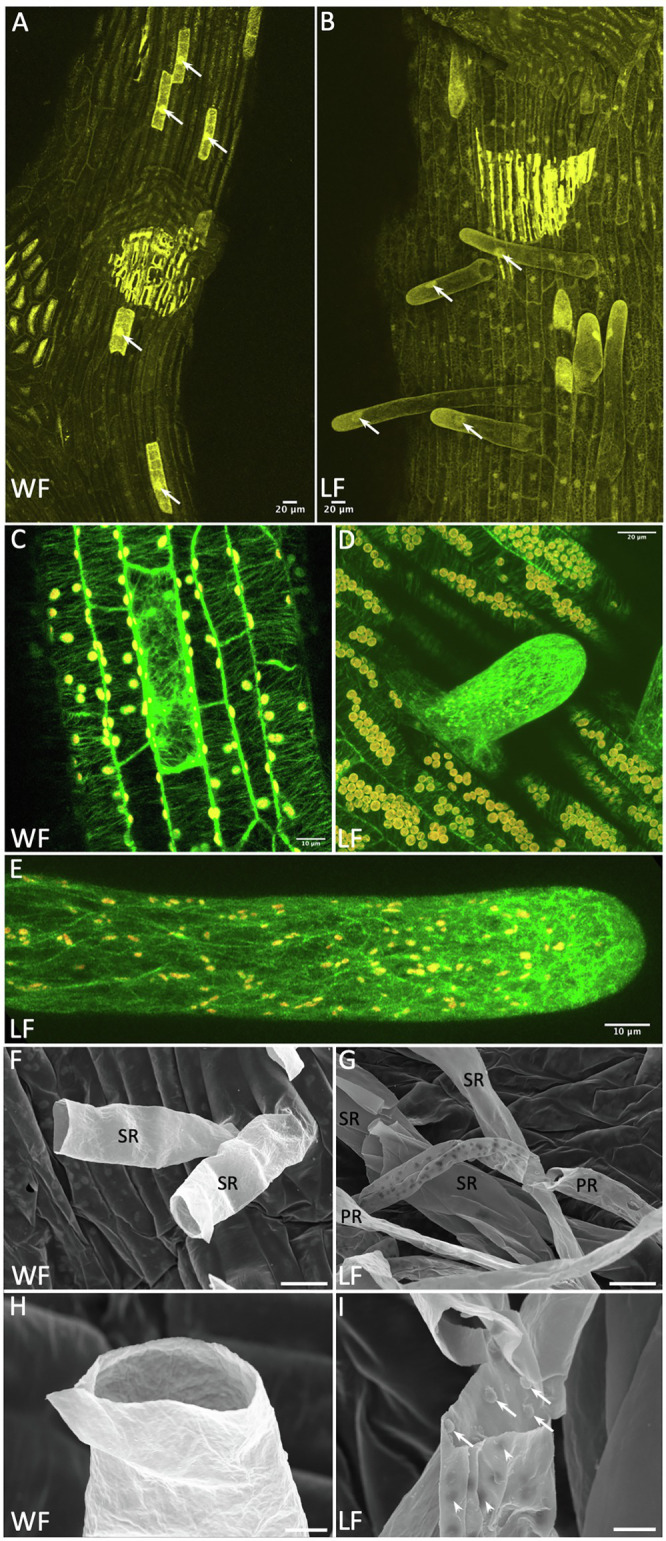
Rhizoid development in the *Riccia fluitans* WF and LF. **(A)** *_pro_*Mp*EF1α*:*mCherry* expression in the ventral WF thallus. Nuclei (arrows) of hypothesized rhizoid initials appear larger than those in surrounding epidermal cells. **(B)** LF mCherry expression, rhizoids with enlarged nuclei are growing out (arrows). **(C)** *_pro_*Mp*EF1α*:*GFP-*Mp*TUB1* expression in ventral WF thallus reveals in some epidermis cells, in contrast to neighboring cells with a parallel, a transversely oriented microtubule array. In outgrowing young **(D)** and older smooth **(E)** LF rhizoids, microtubule density increases heavily toward the tip. **(F–I)** Scanning electron microscopy (SEM) analyses of rhizoids. **(F)** Sections through WF rhizoids reveal only smooth rhizoid (SR) formation. **(G)** Sections through LF rhizoids showed mostly SR and occasionally pegged rhizoid (PR) formation, with variable peg density recognizable from the outside as dark dots. **(H)** Detail of a WF SR with smooth inner and outer surface. **(I)** Detail of a LF PR revealing invaginations on the outer surface (arrowheads) derived from pegs formed as extensions from the inner cell wall, protruding into the rhizoid (arrows). Scale bars: 20 μm, except **(C,E)** 10 μm; **(H,I)** 5 μm.

Using both marker lines, we found that expression levels varied strongly between different ventral epidermal cells. During rhizoid outgrowth, we noticed strong marker gene expression in young WF and LF rhizoids. We therefore assume that the Mp*EF1α* promoter permits stronger gene expression in rhizoid initials and rhizoids with tip growth as compared to other epidermal cell types (Figures 4A–E). Figure 4A shows a ventral WF epidermis, where cells that are strongly expressing mCherry are interspersed between cells with lower mCherry expression levels. The stronger expressing cells also appeared to possess larger nuclei than the surrounding cells. This feature was also observed for rhizoid forming cells in *M. polymorpha* (Duckett et al., 2014), supporting rhizoid initial cell formation in the WF. The situation was similar in the LF, only that here, rhizoid initials are more likely to grow out (Figure 4B). Formation of distinctive rhizoid initial cells is further supported by MpTUB1 analyses. Here, these cells differ from surrounding epidermal cells with highly organized parallel and transversely oriented cortical microtubules, by forming an irregular and intermingled microtubule network (Figure 4C). During rhizoid outgrowth, shown for the LF in Figures 4D,E, microtubules formed a network that became significantly denser toward the cell’s tip. This cytoskeleton arrangement is similar to other tip growth processes, where it is known to mediate signaling and material transport (Geitmann and Emons, 2000).

Complex thalloid liverworts do not only form smooth, but additionally also pegged rhizoids (Duckett et al., 2014). Formation of pegged rhizoids in *R. fluitans* has been controversially described, which might have been affected by restricting analyses to the WF (Klingmüller and Denffer, 1959; Villarreal et al., 2016). Under our cultivation conditions, young LF thalli formed only smooth rhizoids when grown on fresh medium. However, LF plants in older and densely growing cultures developed a few pegged rhizoids. *R. fluitans* rhizoid development was further analyzed by SEM. In the WF, only smooth rhizoid formation was detectable (Figure 4F), which occurred with a strongly reduced frequency compared to the LF. Cross sections of WF rhizoids revealed a smooth inner surface of their cell walls (Figure 4H). Differently, pegged LF rhizoids form small cell wall protrusions extending into the rhizoid cell lumen that are visible from the outside as small, darker invaginations (Figures 4G,I). Both rhizoid types are initiated from cells localized in the central ventral thallus region and most of them are not protected by scales (Figures 1F, 2H,K,N). Together, our data demonstrate the existence of rhizoid dimorphism in the *R. fluitans* LF. However, here, the number of pegged rhizoids is reduced compared to smooth rhizoids and the former were never observed for the WF. Interestingly, WF thalli develop arrested smooth rhizoid initial cells, which might render the WF being capable to quickly respond to an altered, terrestrial environment by formation of anchoring rhizoids.

### Analyses of Cell Division in *Riccia fluitans*

**Figure 5:**
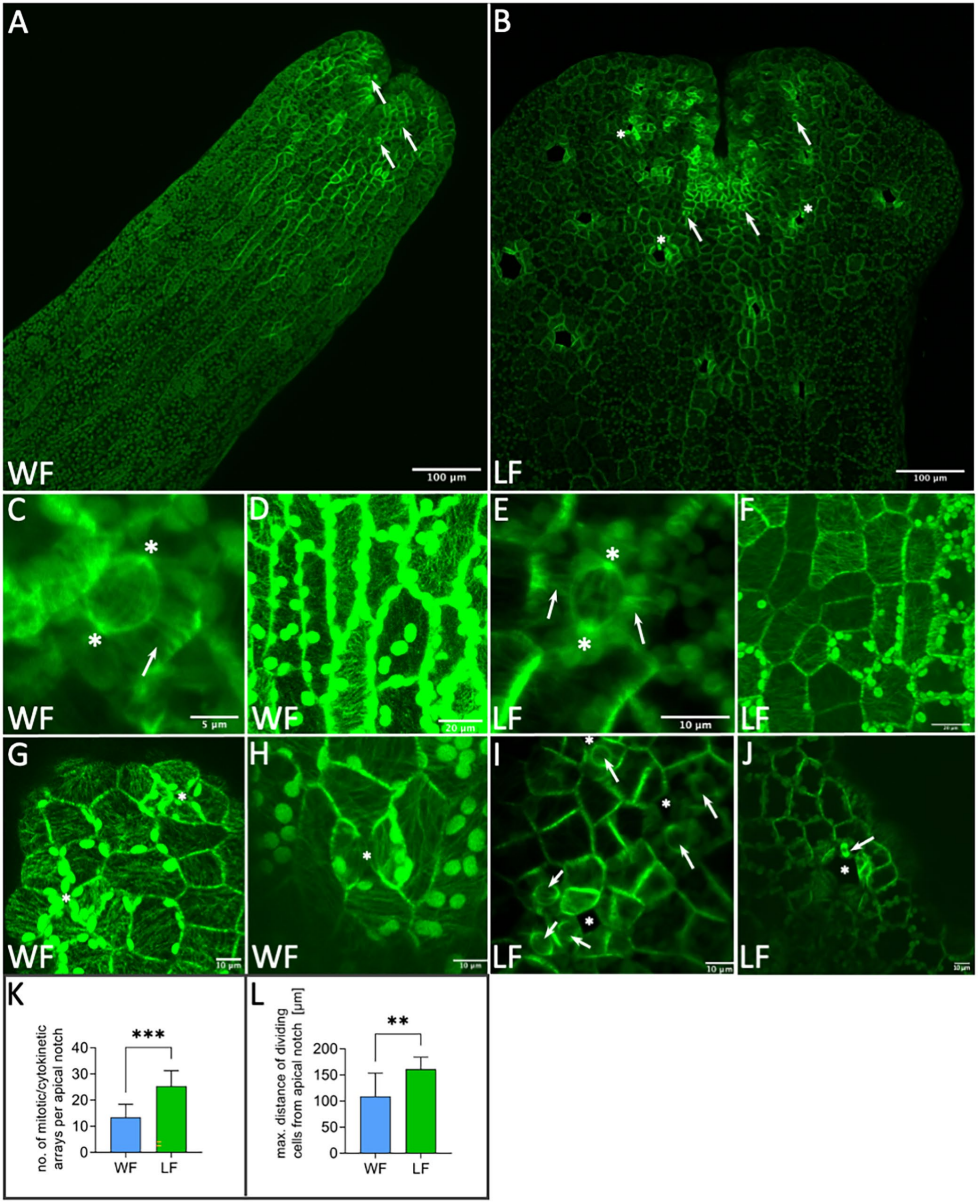
Application of *_pro_*Mp*EF1α*:*GFP*-Mp*TUB1* for analyzing microtubules of *Riccia fluitans*. **(A)** Dorsal view of WF thallus and **(B)** LF thallus tip regions. Arrows in **(A,B)** point to meristematic cell division events. Cell divisions in LF air pore ring cells are marked by asterisks. **(C)** Cell division in WF and **(E)** LF thalli showing polar organizers (asterisks) and preprophase bands (arrows). Highly organized cortical microtubule network in the more elongated WF **(D)** and more squarish LF **(F)** dorsal epidermal thallus cells. **(G)** Air pore invaginations (asterisks) in young WF and **(I)** in LF, near the apical notch region. **(H)** Arrested WF air pore in posterior thallus region. Closure of invaginations by contact of two opposing cell walls is marked with an asterisk. **(I)** Young LF air pore invaginations are surrounded by cells preparing for cell division (arrows), as indicated by polar organizers. **(J)** Older LF air pore (asterisk) in a posterior thallus region showing a mitotic spindle in pore ring cells (arrow). **(K)** Cell division quantification for WF (blue) and LF (green; *n* = 10 apical notches, each form, ^***^p < 0.001). **(L)** Measurement of the maximal distance of cell division from the apical notch for WF (blue) and LF (green; excluding division in air pore ring cells; *n* = 10 apical notches, each form, ^**^p < 0.01). Scale bars: **(A,B)** 100 μm; **(C)** 5 μm; **(D,F)** 20 μm; and **(E,G–J)** 10 μm.

Transgenic *R. fluitans* plants harboring the *_pro_*Mp*EF1α:GFP-*Mp*TUB1* reporter construct were used to investigate cell division processes in WF and LF thalli. Inspecting dorsal overview scans of thallus tips, we noticed that GFP signal distribution was not even. Strongest expression was seen in the vicinity of notches as well as in patches of cells surrounding nascent air pores in LF (Figures 5A,B). We employed the dorsal overview scans to quantify cell division activity in thallus tips (*n* = 10 tips for each form). Around 13.5 ± 4.9 cell divisions were detected per WF thallus tip, while 25.4 ± 5.9 cell divisions were seen in LF tips. This difference was found to be statistically significant (Figure 5K). More cell divisions in LF tips suggested that the LF meristematic region is larger. To support this notion, we used the same data set to calculate the actual size of the meristematic region. This was done by measuring the maximum distance of observable cell divisions to the base of the notch for each form. Cell divisions in LF cells forming air pores were excluded from this measurement. Cell divisions in WF meristematic regions were observed in a radius of 109.4 ± 44.0 μm, while LF meristematic regions showed a radius of 161.0 ± 22.7 μm (Figure 5L). This significant difference in size matches well with the observed 1.5-fold increase of LF thallus width (Figure 1M).

Previous studies in *M. polymorpha* employing GFP-tagged tubulin showed centrosome-like polar organizers and preprophase band formation in cells preparing for mitosis (Buschmann et al., 2016). Indeed, other liverworts are also known to show so-called polar organizers, which resemble algal centrosomes in several aspects (Brown and Lemmon, 2011). The polar organizers of liverworts were previously hypothesized to have derived from the centrosomes of algae (Buschmann and Zachgo, 2016). While pre-mitotic cells of liverworts simultaneously form preprophase bands, the latter structures are lacking in algae, with the possible exception of the isthmus band of microtubules seen in some Zygnematophyceae (Buschmann and Zachgo, 2016). We therefore asked whether these mitotic and cytokinetic microtubule arrays differ between the WF and LF thalli. As expected, we observed polar organizers and preprophase bands in the LF (Figure 5E). Similarly, these mitotic arrays were also seen in the WF (Figure 5C), suggesting that the mechanism of *R. fluitans* cell division is not affected by differences in growth conditions. Moreover, once the epidermal thallus cells have moved out of the region of ongoing cell division (here imaged at 400 μm distance from the base of the notch), WF and LF both form highly organized cortical microtubule arrays, with stronger elongated epidermal cells in WF compared to LF plants (Figures 5D,F). Similar differences in cell size were also seen in our SEM-based cell size quantifications (Figures 1K,L,N).

Cells of WF air pores rarely showed any sign of cell division, likely due to arrest of the four pore ring cells at an early stage (Figures 5A,G,H). In contrast, mitotic and cytokinetic structures were frequently seen in pore ring cells surrounding young and as well as older LF air pores (Figures 5B,I,J), emphasizing that an increase of pore ring cell number contributes to the enlargement of LF pore openings.

### Cell Wall and Cuticle Analyses in *Riccia fluitans* WF and LF

**Figure 6:**
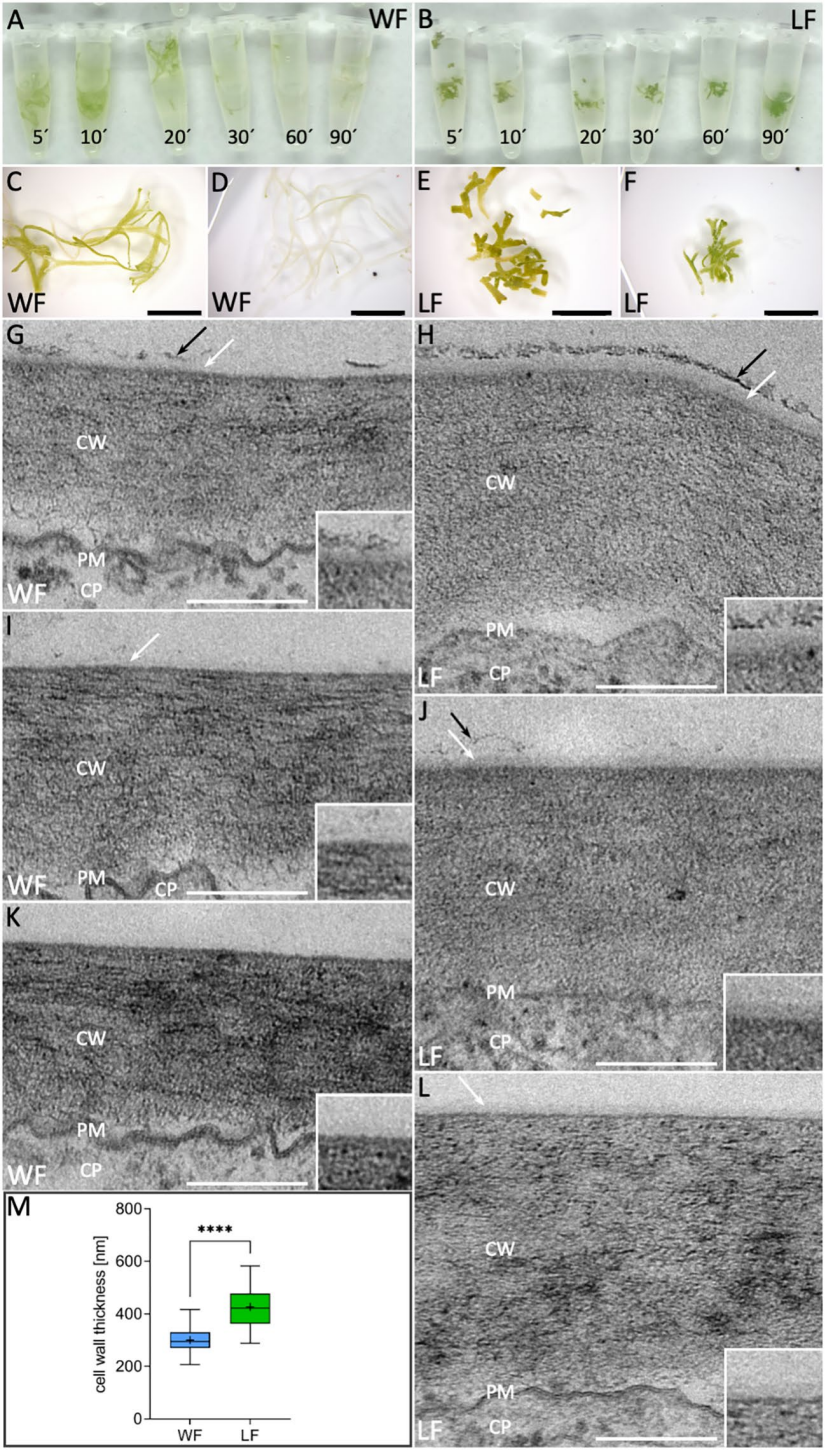
Cell wall and cuticle analyses in *R. fluitans*. **(A)** WF and **(B)** LF thalli after bleaching in ethanol for 5, 10, 20, 30, 60, and 90 min. **(C,E)** WF/LF thalli after 5 min treatment and **(D,F)** 120 min. **(G,I,K)** Cross sections of outer, periclinal cell walls of dorsal WF epidermal cells reveal variable, weak cuticle formation (white arrows). Cuticle is rarely topped with a fragmented additional darker layer (black arrows), likely representing epicuticular waxes **(G)**. **(H,J,L)** Corresponding LF cell walls are thicker and cuticle formation is more consistent (white arrows). A darker, patchy putative wax layer of varying thickness was more frequently observed (**H,J**, black arrows), but could also be absent **(L)**. Insets in **(G–L)** show details of outermost cell wall zones, **(K)** and **(J)** are the most representative images for WF and LF, respectively. CW, cell wall; PM, plasma membrane; and CP, cytoplasm. **(M)** Quantification of outer cell wall thickness in WF (blue) and LF (green) dorsal epidermal thallus cells (WF, *n* = 69 cells of three individual thalli; LF, *n* = 74 cells of three individual thalli; ^****^p < 0.0001). Scale bars: **(C–F)** 5 mm; **(G–L)** 200 nm.

The cuticle, composed of cutin and associated hydrophobic waxes, prevents water loss resulting in dehydration (Yeats and Rose, 2013). Cuticle and cell wall permeability differences can be monitored by chlorophyll release under ethanol exposure (Lolle et al., 1997). We compared WF and LF thallus surface permeability by imbibing plants in ethanol and monitored chlorophyll bleaching over a time course of 120 min (Figures 6A,B). No obvious differences of WF and LF thalli were observable after 5 min bleaching (Figures 6C,E), but then WF plants revealed a quicker and stronger bleaching effect such that plants were translucent after 120 min (Figure 6D). Contrarily, even after 120 min, LF thalli showed no obvious color changes (Figure 6F). Compared to the WF, LF plants thus possess a reduced surface permeability.

To investigate differences of WF and LF cell walls and cuticle layers in more detail, we conducted TEM analyses of the outer periclinal walls of dorsal epidermal cells. Here, the cell wall of both forms is visible as a broad gray layer with variable striated patterns and separated from the cytoplasm by a dark undulated line, representing the plasma membrane (PM; Figures 6G–L). The thickness of the *R. fluitans* cell wall seemed to vary between the WF (Figures 6G,I,K) and LF (Figures 6H,J,L) and was therefore quantified in differentiated cells from posterior thallus regions. Average WF cell wall thickness was 300 ± 47 nm compared to 427 ± 73 nm in LF cell walls (Figure 6M). The determined significant 1.4-fold difference in cell wall thickness confirmed first impressions from the TEM analyses. In WF and LF TEM images, the cuticle is visible as thin, translucent layer with variable appearance overlaying *R. fluitans* thallus epidermis cell walls (Figures 6G–L and insets). For LF cells, a distinct cuticle of varying thickness and relatively high contrast was detected in all sections (Figures 6H,J,L), with Figure 6J showing the most representative example. Occasionally, the cuticle was overlayed by a patchy darker layer, which most likely represents epicuticular waxes (Figures 6H,J). The structure of this wax layer was, however, not as massive and dense as observed in air pores (Figure 3Jc) and appeared more pronounced on LF than WF epidermal walls (Figures 6G,H). For the WF, the most representative example is shown in Figure 6K, where even the cuticle seems to be entirely missing. However, occasionally, also thinner cuticle layers with weaker contrast were observed (Figures 6G,I). Our cell surface analysis thus demonstrates differences between the *R. fluitans* WF and LF surface permeability, likely mediated by changes of epidermal cell walls, cuticle and epicuticular wax layers in adaptation to the two different growth environments.

## Discussion

*R. fluitans* is an amphibious liverwort in the most species-rich family within the Marchantiopsida, the Ricciaceae (Müller, 1941; Klingmüller and Denffer, 1959; Söderström et al., 2016). We took advantage of its capability to adjust its morphology and growth to two different environments and focused in this study on investigating air pores, rhizoids, cell walls and cuticle in the WF and LF. These structures were crucial for the evolution of the land plant flora, as they mediate gas exchange, water and mineral uptake from soils and protection against drought.

### Plasticity of *Riccia fluitans* Air Pore Development

Initiation of air pore formation starts in apical notch areas of the LF and also of the WF, where an invagination is formed by four pore ring cells, supporting earlier observations from Leitgeb (1880) and Klingmüller and Denffer (1959). In the WF, the cell walls of two opposing pore ring cells stay in contact. Small pore gaps can be formed, which are closed by deposition of an extracellular mass and formation of protrusions from the two opposing pore ring cells. Pore development is arrested and the four WF pore ring cells do not divide. Differently, in the LF an increase from four to sometimes up to 10 pore ring cells can occur and cell divisions likely contribute to enlarge the pore opening. Extracellular masses, possibly mucilage material or waxes, as well as central connecting bar-shaped structures that might be composed of cell wall material finally disappear, generating an open pore capable to mediate gas exchange.

Intercellular spaces can be generated in plants by lysogeny, spatially limited cell death, or by schizogeny, where cells are separated by differential growth (Ishizaki, 2015). Here, we show that formation of *R. fluitans* intercellular spaces is mediated by a schizogenous mechanism, as described for the genus *Marchantia* (Ishizaki, 2015). *M. polymorpha* forms complex air pores that are built by four concentric rings of cells forming a barrel-shaped structure. Differently, *R. fluitans* pores are less complex and were categorized as simple openings without ring formation (Villarreal et al., 2016). Our studies demonstrate the presence of at least four pore ring cells, grouping them into the category of simple pores with one ring of cells. Assimilatory, chlorophyllous filaments formed in *M. polymorpha* air chambers are absent in *R. fluitans*. Loss of function of Mp*NOP1* impairs air pore and air chamber formation in *M. polymorpha* (Ishizaki et al., 2013). *M. polymorpha* plants with reduced activity of the Mp*WIP* transcription factor initiate air pore formation with four pore cells and a central small schizogenous opening. However, further development is then disrupted (Jones and Dolan, 2017), thereby resembling arrested *R. fluitans* WF pores. It will thus be interesting to investigate if the activity of the *R. fluitans* Mp*WIP* and Mp*NOP1* orthologs are affected by different environments.

Air pores are always open in liverworts and cannot be closed as stomata in other land plants, therefore, water could enter the pores by capillary attraction and block them (Schönherr and Ziegler, 1975). A comprehensive study on liverwort air pore geometry reported the occurrence of hydrophobic cuticular layers on pore ring cell ledges in 12 of the 14 investigated species. These ledges extend toward the center of the pore and can thereby likely prevent water entry into air pores and intracellular thallus spaces (Schönherr and Ziegler, 1975). *R. fluitans* does not form such pore ring cell ledges, but we detected deposition of a layer in LF air pores that likely represent epicuticular waxes. This massive layer starts on the LF pore ring cells directly behind the narrowest pore position and disappears on the cell below the pore ring cell. Thus, *R. fluitans* also seems to exploit a protective hydrophobic layer mechanism, where epicuticular waxes restrict water entrance into air chambers.

### Plasticity of Dimorphic *Riccia fluitans* Rhizoid Development

Besides schizogenous air pore formation, also development of dimorphic rhizoids is a trait that evolved exclusively in complex thalloid liverworts (Villarreal et al., 2016). Under our LF growth conditions, *R. fluitans* produces besides a high number of smooth rhizoids also a few pegged rhizoids with varying numbers of pegs, cell wall protrusions reaching into the rhizoid lumen. After extended WF growth periods, formation of solely smooth rhizoids was also detectable. Whereas in other Marchantiales pegged rhizoids emerge below scales and smooth rhizoids on scale free thallus regions (McConaha, 1941), we observed a dispersed pattern of smooth and pegged rhizoid formation between and beneath the scales in *R. fluitans*. In *M. polymorpha*, pegged rhizoids are formed at larger numbers and converge during elongation into strands running parallel to the ventral thallus surface for water conductance (McConaha, 1941; Cao et al., 2014; Duckett et al., 2014). The diversity in Marchantiales rhizoid formation and patterning likely reflects adaptations to different ecological niches, with *R. fluitans* WF not requiring an external water capillary system and LF thriving with only a reduced system, given damp shore regions of ponds and muddy floodplains after water withdrawal as typical habitats (Borovichev and Bakalin, 2016).

Notably, GFP-MpTUB1 analyses showed that rhizoid initials are very likely also formed in the WF, where further outgrowth is then, however, arrested. Thalli that were exclusively grown on solid medium as well as fully adapted WF plants at 15 DAT to solid medium, all form tip-growing rhizoid cells, starting proximal to the first mature scale. Differently, in an early transition phase, at 3 DAT and 6 DAT, tip-growing cells were detected in further proximal regions, suggesting they derived from reactivation of arrested WF rhizoid initial cells. Our data thus suggest that WF rhizoid initial cells can respond to environmental conditions and either arrest further development in water, or given terrestrial conditions, resume rhizoid development by initiating tip growth. mCherry expression in nuclei indicated that the nuclei in WF initial cells as well as outgrowing LF rhizoids appeared larger than in nuclei of surrounding epidermal cells. *M. polymorpha* smooth rhizoids form enlarged nuclei with numerous nucleolar fragments, which are likely generated by endoreduplication events and might contribute to extreme tip growth of up to 3 cm in length (Duckett et al., 2014). *M. polymorpha* rhizoid development and patterning is controlled by Mp*RSL1* (Proust et al., 2016), which is directly repressed by the miRNA Mp*FRH1* (Honkanen et al., 2016; Thamm et al., 2020). Future investigations of the regulatory Mp*RSL1*/Mp*FRH1* activities in *R. fluitans* can shed light on whether and how these regulators contribute to mediate the here observed adaptations. A high variability of miRNA occurrence in different liverwort lineages (Alaba et al., 2015) might have contributed to diversify miRNA regulatory functions for adjustments to altered environmental conditions.

### Cell Wall and Cuticle Adaptations to a Terrestrial Life Style

The cell wall and cuticle are extracellular structures that played critical roles in the adaptation of plants to terrestrial habitats, as they regulate water exchange, provide UV protection and mechanical support, as well as biotic stress protection (Sørensen et al., 2010; Philippe et al., 2020). Chlorophyll clearing experiments suggested differences between these structures, mediating a reduced LF surface permeability compared to the WF. TEM analyses demonstrate that in contrast to the WF, the LF develops a 1.4-fold thicker cell wall and we observed a more pronounced cuticle and epicuticular wax layer formation on dorsal epidermal cells. In the WF, a cuticle was often missing or only visible as a thin, weakly contrasted layer and epicuticular layer formation was strongly reduced.

Similar to other bryophytes, a clear subdivision of the cuticle into different sub-layers, as observed in tracheophytes, was not detected (Guzmán et al., 2014; Shimamura, 2016; Renault et al., 2017; Koshimizu et al., 2018; Guignard, 2021). Three complex hydrophobic biopolymers contribute to permeability control in vascular plants: cutin, suberin, and lignin. Their monomeric precursors are ancestral to land plants and some of these components already exist in algae (Niklas et al., 2017; Koga et al., 2021). A pre-lignin-pathway was identified in *Physcomitrium patens* as well as the biosynthetic pathway for cuticle formation (Buda et al., 2013; Renault et al., 2017; Kong et al., 2020). In the liverwort *M. polymorpha*, the Mp*SBG9* transcription factor is a key regulator of cuticle biosynthesis and controls surface permeability (Xu et al., 2021). Additionally, lineage-specific compounds contribute to adaptive cell wall remodeling processes, as revealed by recent *M. polymorpha* cell wall proteome analyses (Kolkas et al., 2021). It was also shown that angiosperms can adapt surface layers in response to an altered environment. Terrestrial and aquatic leaves of *Rumex palustris* differ in cell wall and cuticle formation, where leaves develop significantly thinner cuticle layers and cell walls when grown submersed in water (Mommer et al., 2005), resembling our observations. Additionally, only chloroplasts of terrestrial *R. palustris* leaf cells are orientated toward intercellular spaces. Similarly, chloroplasts of the *R. fluitans* LF preferentially accumulate on cell walls facing air chambers. As suggested for *R. palustris* (Mommer et al., 2005), differences in subcellular chloroplast localization might optimize access to variable CO_2_ availabilities in the two growth forms of *R. fluitans*.

### *Riccia fluitans*: An Ideal Model System to Study Water to Land Adaptations

Amphibious plants enable to study the impact of environmental changes on essential adaptive traits encoded by a single genotype. The transition of the *R. fluitans* WF to LF is accomplished within 15 days by then forming mature, open LF air pores, functional rhizoids and an expanded thallus width, mediated by more cell divisions in apical notch areas. An established *R. fluitans* transformation protocol, applied here to extend morphological characterizations employing MpTUB1 and mCherry reporter lines, combined with an easy cultivation procedure and a genome size of around 1 GB (Temsch et al. 2010), altogether provide an ideal basis for this amphibious liverwort being used in future analyses. Recently, studies of amphibious angiosperms gave insight into molecular frameworks regulating heterophylly (Nakayama et al., 2017; Kim et al., 2018; Koga et al., 2021). The developmental plasticity of *R. fluitans*, belonging to the first lineage that separated from a common ancestor of embryophytes, can now be exploited to shed light on evolutionary trajectories of crucial molecular adaptations during the conquest of land.

## Data Availability Statement

The original contributions presented in the study are included in the article/supplementary material, further inquiries can be directed to the corresponding author.

## Author Contributions

FA, LW, and HB: experimental investigations. KE: supervision of TEM and light microscopy. FA: original draft preparation. SZ: conceptualization, supervision, and final manuscript writing with support of all authors. All authors contributed to the article and approved the submitted version.

## Funding

We are grateful for funding by the DFG in the framework of the SPP2237 MadLand (KE: EH 372/1-1; SZ: SZ 259/10-1; and HB: BU 2301/6-1) and to SZ by the SFB944/P13.

## Conflict of Interest

The authors declare that the research was conducted in the absence of any commercial or financial relationships that could be construed as a potential conflict of interest.

## Publisher’s Note

All claims expressed in this article are solely those of the authors and do not necessarily represent those of their affiliated organizations, or those of the publisher, the editors and the reviewers. Any product that may be evaluated in this article, or claim that may be made by its manufacturer, is not guaranteed or endorsed by the publisher.
